# Artificial intelligence accelerates the mining of bioactive small molecules from human microbiome

**DOI:** 10.1002/ctm2.1011

**Published:** 2022-08-15

**Authors:** Yuwei Zhang, Pengwei Li, Yue Ma, Jun Wang, Yihua Chen

**Affiliations:** ^1^ State Key Laboratory of Microbial Resources, Institute of Microbiology Chinese Academy of Sciences Beijing China; ^2^ College of Life Sciences University of Chinese Academy of Sciences Beijing China; ^3^ CAS Key Laboratory of Pathogenic Microbiology and Immunology, Institute of Microbiology Chinese Academy of Sciences Beijing China

1

**FIGURE 1:**
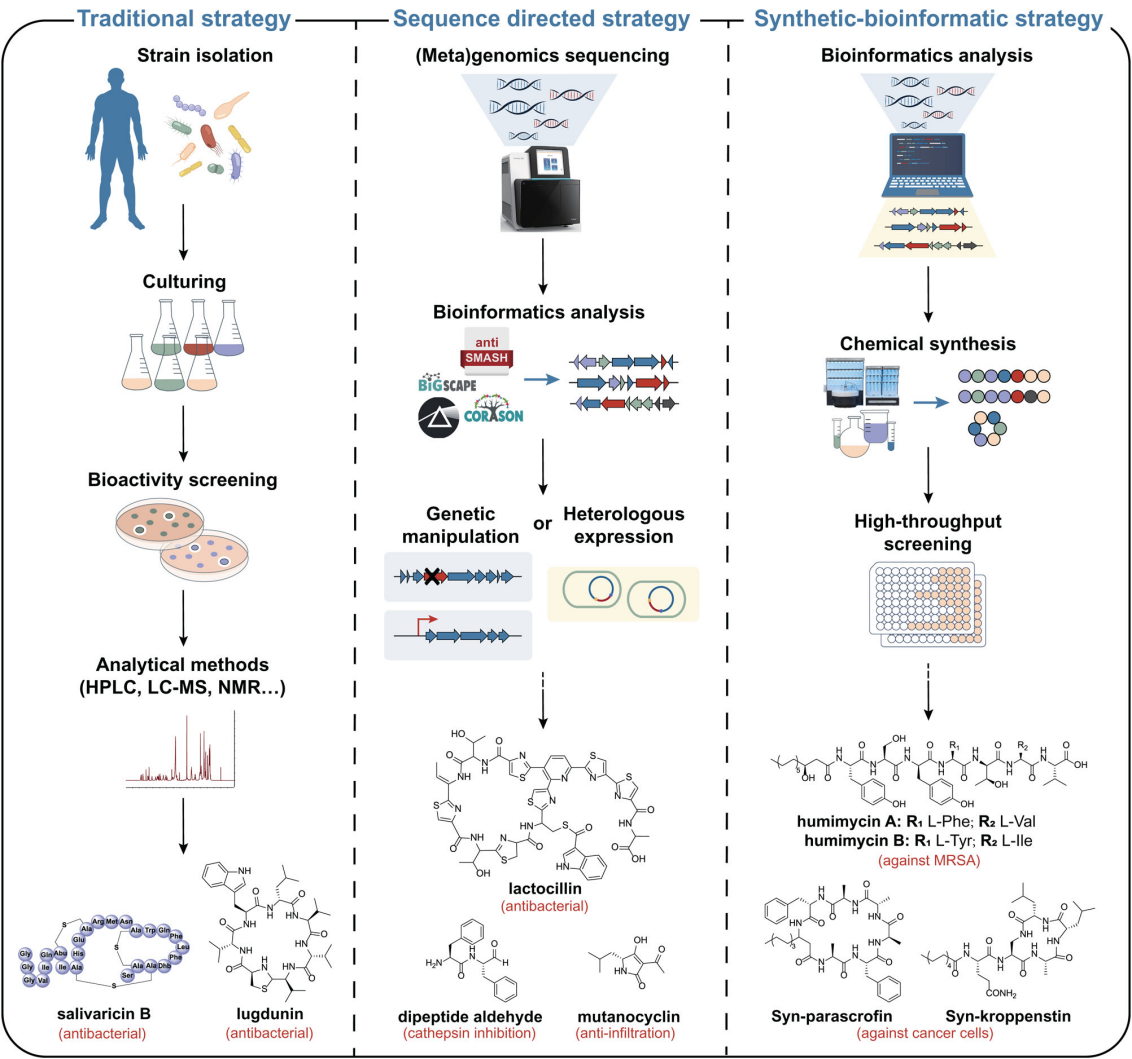
Flowcharts of mining bioactive small molecules from human microbiota by different strategies. Left, the traditional strategy relies on the cultivation of microbes, bioactive molecules were discovered through activity‐oriented isolation process. Middle, the sequence‐directed strategy predicts biosynthetic gene clusters (BGCs) through bioinformatics analysis and obtains the products with the help of genetic manipulation or heterologous expression. Right, the synthetic‐bioinformatic strategy generates the small molecules, which are designed by analysis on certain types of BGCs, by chemical synthesis.

With the threat of increasing antibiotic‐resistant pathogens and invasive infection and mortality worldwide, the discovery of new and potent pharmaceuticals is of great urgency. Human microbiota provides a tremendous arsenal to discover metabolites with promising antibiotic properties. By applying innovative culture methods for newly isolated microbes or adopting activity‐oriented experimental discovery processes, scores of natural products with antibacterial activity have been identified from the human microbiota, for example ribosomally synthesized and post‐translationally modified peptides including salivaricin,^1^ and non‐ribosomal peptide lugdunin^2^ (Figure 1). Nonetheless, this approach faces challenges in obtaining natural products produced by unculturable microbes or undetectable under experimental conditions.

With the rapid development of DNA sequencing technology in recent decades, a vast amount of (meta)genomic data from human microbiota has become increasingly available, providing enticing opportunities to unveil the ‘dark matters’ hidden in their genomes. Biosynthetic genes responsible for the synthesis of bioactive natural products are usually clustered in microbial genome and have different characteristic sequences, making it possible to predict the biosynthetic gene clusters (BGCs) through *in silico* approach relying on sequence signatures. In the past few years, increasing bioinformatics tools, including antiSMASH, ClusterFinder and BAGEL4, which mainly utilize BLAST (basic local alignment search tool) searches or pHMMs (profile hidden Markov models), were well developed for the analysis of BGCs.^3^ With the assistance of these tools and various genetic manipulations as well as heterologous expression systems, a number of natural products have been obtained and characterized to give more insights into their ecological roles and interaction with hosts (Figure 1). Take mutanocyclin, which is secreted by *Streptococcus mutans* strains that can cause tooth caries, as an example, this molecule was discovered by the expression of its BGC in a model strain *S. mutans* UA159.^4^ Further investigation revealed that mutanocyclin has immunomodulatory activity and can suppress the filamentous growth of *Candida albicans*, indicating its role in helping the *S. mutans* host withstand the pressures from human immune system and the other microbes. Lactocillin^5^ with antibacterial activity and dipeptide aldehydes^6^ with cathepsin inhibitory activity also possess similar discovery paths. In addition, bioinformatic‐inspired structure prediction combined with chemical synthesis, namely syn‐BNP (synthetic‐bioinformatic natural products), was established to dig out novel peptides derived from human‐associated bacteria, such as humimycin,^7^ syn‐parascrofin and other syn‐peptides^8^ (Figure 1). This pipeline circumvented the requirements of microbial culture and BGC expression, thus greatly improving the efficiency of mining bioactive natural products or their analogues. However, methods based on BGCs prediction have certain limitations in the accuracy of the predicted structures, more effective and targeted screening methods are also needed to match the growth rate of (meta)genomic data. Therefore, more promising pipelines and strategies are of great need to expedite the exploitation of novel bioactive natural products from the immense untapped human microbiome.

**FIGURE 2:**
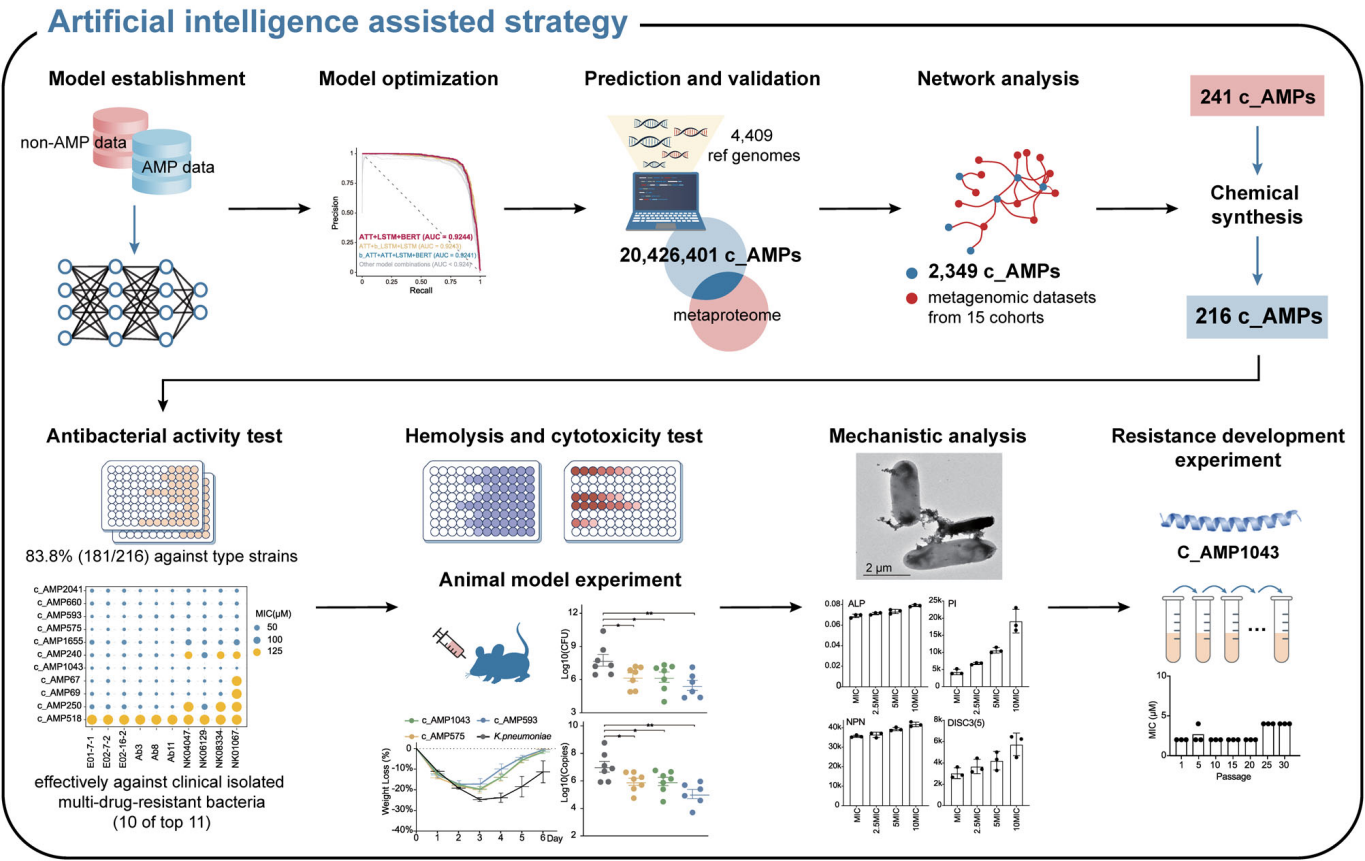
Workflow of the artificial intelligence (AI)‐assisted strategy for the discovery of antimicrobial peptides (AMPs) from human microbiome

More recently, artificial intelligence (AI) has been applied to the discovery of antibiotics, some molecules with antibiotic activity were successfully screened from the existing compound library or directly designed,^9^ which provides a guarantee for the feasibility of mining in human microbiome using AI‐assisted approach. Antimicrobial peptides (AMPs) are considered promising antimicrobial agents due to their wide bioactivity spectra, low tendency to induce resistance and availability through chemical synthesis. However, its relatively short sequence length and high diversity hinder the application of current mining methods. In our work,^10^ multiple neural network models (NNMs), including attention, long short‐term memory (LSTM) and Bidirectional Encoder Representations from Transformers (BERT), were combined to form a pipeline for AMPs mining, which achieved 91.31% precision and maintained a low false‐positive rate (Figure 2). Using this pipeline, a total of 20 426 401 AMPs were predicted from 4409 qualified representative genomes from human microbiome. To ensure that the obtained AMPs are expressed in human body, we performed cross‐validation with metaproteomic data and refined the range of candidate AMPs to 2349. Considering the potential negative correlations between functional AMPs and the bacteria they can inhibit, AMP correlation networks were constructed using metagenomic datasets from 15 independent cohorts, and the list of candidate AMPs was further narrowed down to 241. Subsequently, 216 successfully synthesized AMPs were examined in the initial antibacterial activity test, and results showed a positive rate up to 83.8% (181/216). In addition, most of the 181 AMPs showed less than 40% identity with previously reported antibacterial AMPs, indicating that our pipeline was able to identify novel AMPs based on internal relationships of amino acids in the sequences instead of relying on sequence similarity. The top 11 AMPs were further tested and 10 AMPs showed high antibacterial activity against clinically isolated multi‐drug‐resistant bacteria. Among them, three AMPs with relatively low haemolysis and cytotoxicity were tested on mouse models infected with *Klebsiella pneumoniae* and showed significant therapeutic effects. Moreover, the resistance development experiment of one AMP against *Escherichia coli* DH5α showed no observed resistance after 30‐day passaging, which further demonstrates the therapeutic potential of AMPs we identified.

To sum up, the high positive rate, effectiveness and novelty of the AMPs we identified exhibit the high efficiency of AI‐assisted approach in the mining of bioactive molecules from human microbiome. Besides the application of NNMs, *in silico* high‐throughput and targeted screening using metaproteomic data and association network analysis significantly reduced the excessive workload and cost of traditional in vitro experiments. As shown in our results, the long‐term symbiosis and competition existing in human microbiota have derived bioactive molecules like AMPs with clinical application potential. It is worth noting that with different sources of training data and different strategies in the *in silico* screening, the pipeline we developed can also be applied for the exploration of different types of natural products with other desired activities. With the acquisition of more omics data (including clinical data) and the progress of sequencing technology at the strain‐level resolution, AI‐assisted mining strategy can effectively accelerate the high‐throughput and goal‐oriented screening process that was time‐consuming in the past, which can be very useful in the discovery of druggable molecules from microbes in different habitats.
